# Cognitive Domain Impairments in Chronic Painful Temporomandibular Disorders: Associations With Pain Intensity, Hypervigilance and Catastrophising: A Cross‐Sectional Analysis

**DOI:** 10.1111/joor.70064

**Published:** 2025-09-24

**Authors:** Julio Ruiz‐Marrara, Luiza Guilherme Antunes, César Bataglion, Maria Helena Fernandes, Melissa de Oliveira Melchior, Laís Valencise Magri

**Affiliations:** ^1^ Faculdade de Medicina Dentária da Universidade do Porto Porto Portugal; ^2^ Departamento de Prótese e Materiais Dentários Faculdade de Odontologia de Ribeirão Preto, Universidade de São Paulo Ribeirão Preto/São Paulo Brazil; ^3^ Departamento de Odontologia Restauradora Faculdade de Odontologia de Ribeirão Preto, Universidade de São Paulo Ribeirão Preto/São Paulo Brazil

**Keywords:** chronic orofacial pain, cognitive impairment, pain catastrophising, pain hypervigilance, temporomandibular disorder

## Abstract

**Background:**

This study aimed to investigate the associations between pain intensity, hypervigilance and catastrophising with specific cognitive domains in patients with chronic painful temporomandibular disorders (TMD), focusing on identifying cognitive deficit domains.

**Methods:**

A cross‐sectional study was conducted with a sample of 80 participants (41 chronic painful, 39 controls). TMD was diagnosed according to the Diagnostic Criteria for TMD. Cognitive performance was assessed using the Montreal Cognitive Assessment (MOCA), while pain‐related factors were evaluated using the Pain Vigilance Awareness Questionnaire, the Pain Catastrophizing Scale and Pain Intensity. Statistical analyses included independent *t*‐tests to compare group means, followed by multiple regression analysis to examine the associations between cognitive domains and pain‐related variables.

**Results:**

Compared to controls, TMD patients showed significantly lower MOCA scores in visuospatial/executive functioning (mean difference = 3.3, *p* = 0.03), attention (mean difference = −2.5, *p* < 0.002) and memory (mean difference = −1.8, *p* = 0.001). Higher pain intensity was significantly associated with visuospatial/executive deficits (*β* = −0.34, 95% CI: −0.50 to −0.18; *p* < 0.001), while catastrophising was strongly linked to reduced attention (*β* = −0.05, 95% CI: −0.08 to −0.02; *p* < 0.001). Hypervigilance showed a significant negative correlation with memory performance (*β* = −0.04, *p* = 0.003).

**Conclusions:**

Our study reveals that chronic painful TMD is associated with some cognitive impairments, particularly in visuospatial/executive functioning, attention and memory. Elevated pain intensity and catastrophising emerged as the most impactful factors, closely linked to deficits in these cognitive domains. These findings identify executive control, attention and memory as the cognitive domains most vulnerable to the influence of chronic pain in TMD patients.

**Significance:**

This study provides novel insights into the cognitive impairments associated with chronic painful TMD. By demonstrating strong associations between pain intensity, hypervigilance and catastrophising with deficits in executive function, attention and memory, our findings highlight the cognitive burden of chronic TMD. These results underscore the necessity of integrating cognitive and psychological assessments into clinical management, fostering a more comprehensive approach to treatment that extends beyond pain relief to improving cognitive function and overall quality of life.

## Graphical abstract

Cognitive impairments in chronic painful temporomandibular disorders (TMD) are linked to higher pain intensity, hypervigilance and catastrophising. In a sample of 80 participants, chronic painful TMD was associated with deficits in visuospatial/executive functioning, attention and memory. Multiple regression analyses revealed that pain‐related variables significantly contributed to these cognitive domain alterations.

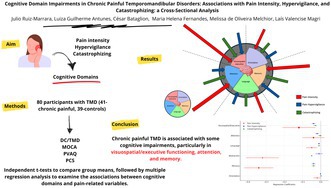

## 1 Introduction

Temporomandibular disorders (TMD) are conceptualised as a complex array of signs and symptoms indicative of a musculoskeletal pain syndrome, often associated with multisystemic alterations. These include behavioural modifications, shifts in emotional state and disruptions in social interactions, recognised as manifestations of central nervous system dysregulation [1, 2, 3]. Key predictors for TMD onset encompass comorbid conditions, non‐painful orofacial symptoms (such as self‐reported parafunctional behaviours), a high frequency of somatic symptoms, poor sleep quality and both genetic and epigenetic factors [1, 2].

Emerging research highlights the relationship between chronic pain and cognitive deficits, including impairments in memory, attention and executive functioning, which are commonly reported in patients with TMD [4, 5]. These cognitive impairments are thought to arise due to the intense cognitive demands of pain processing, which diverts resources away from other cognitive functions, particularly in the prefrontal cortex where pain and executive processes are managed [4, 5, 6].

These cognitive deficits appear to be linked to several neurophysiological and neurochemical changes induced by chronic pain, affecting both the structure and function of the brain. One central mechanism proposed is the continuous and abnormal activation of the hypothalamic–pituitary–adrenal (HPA) axis, which elevates cortisol levels and leads to neurotoxicity [4, 7, 8]. Hyperactivity in areas such as the dorsolateral prefrontal cortex and hippocampus demands excessive cognitive resources for pain modulation, leaving fewer resources available for executive functions, planning and memory [4, 9].

Pain‐related psycho‐cognitive aspects, such as pain catastrophising and hypervigilance, also contribute to the negative impact of chronic pain on cognitive functions. Studies suggest that the impact of chronic pain on cognition appears to be mediated by a combination of changes in the nervous system, including cortisol‐induced neurotoxicity, neuroinflammation, central sensitisation and emotional overload [5, 6, 8].

Emerging evidence has suggested that specific pain‐related psychological constructs may be differentially associated with distinct cognitive domains. Pain intensity has been strongly linked to impairments in visuospatial and executive functions, likely due to the increased demand for attentional resources and altered activity in prefrontal cortical networks involved in pain modulation and cognitive control [4, 5]. Hypervigilance to pain, characterised by excessive attention to pain‐related stimuli, has been associated with reduced memory performance, possibly due to competition for working memory resources and heightened stress responses that interfere with encoding and retrieval processes [10]. Similarly, pain catastrophising has been implicated in diminished attentional control, as it amplifies emotional arousal and disrupts executive attentional networks, leading to difficulties in sustaining attention and filtering irrelevant stimuli [11, 12].

The use of the Montreal Cognitive Assessment (MoCA) in chronic pain research has shown promise in identifying domain‐specific cognitive impairments across various conditions, including fibromyalgia, chronic back pain and migraine [4, 12]. These studies often report consistent deficits in attention, memory and executive functions, supporting the rationale for exploring similar domains in chronic painful TMD.

Chronic painful TMD is highly prevalent and can significantly impair patients' quality of life by affecting multiple dimensions, including cognitive functioning, emotional regulation and physical health. The interplay between pain perception, such as pain hypervigilance and catastrophising, and cognitive performance has been increasingly recognised as a critical area of study [6, 8, 13]. However, there is a paucity of research specifically examining the impact of these pain‐related variables on cognitive domains such as attention, memory and executive function in individuals with chronic painful TMD. The aim of this study is to investigate the associations between pain intensity, hypervigilance and catastrophising and cognitive domains in patients with chronic painful TMD. Specifically, we hypothesise that: higher pain intensity will be associated with deficits in visuospatial/executive functioning, increased catastrophising will correlate more strongly with reduced attention, and hypervigilance will be associated with poorer memory performance.

## 2 Materials and Methods

### 2.1 Study Design

This is a cross‐sectional study designed to investigate the associations between cognitive domains and pain‐related variables in patients with chronic painful TMD.

### 2.2 Ethical Aspects

This study was approved by the Research Ethics Committee of the School of Dentistry of Ribeirão Preto at the University of São Paulo, under CAAE number 03383218.7.0000.5419, ensuring that all ethical standards for research involving human participants were thoroughly met. Ethical approval followed a rigorous evaluation to protect participant welfare and uphold principles of confidentiality, informed consent and voluntary participation. Participants were fully informed about the study's objectives, procedures, potential risks and benefits, and they provided written informed consent prior to participation. This protocol aligns with ethical guidelines set forth in the Declaration of Helsinki and adheres to national standards established by Brazil's National Health Council, ensuring that the rights and dignity of all participants are respected throughout the research process.

### 2.3 Sample Composition

The sample size was estimated based on prior studies that evaluated cognitive performance using the MoCA in populations with chronic pain [4, 11, 14]. These studies reported mean group differences in MoCA total or domain‐specific scores between 2.0 and 3.0 points, with standard deviations ranging from 3.0 to 3.5. For this study, we considered a mean difference of 2.5 points in total MoCA score between the TMD and control groups as clinically relevant. Assuming a standard deviation of 3.5, this corresponds to an expected effect size of approximately 0.71 (Cohen's *d*), which represents a moderate‐to‐large effect. Based on a two‐tailed independent samples *t*‐test, a significance level of 5% (*α* = 0.05), and a statistical power of 80% (1–*β* = 0.80), the minimum sample size required was 35 participants per group. To account for potential dropouts, we aimed to include at least 39 participants per group. The sample size calculation was performed using G*Power software (version 3.1.9.2), and was based on detecting differences in global cognitive functioning (MoCA total score) in relation to pain‐related psychological factors, particularly pain catastrophising and hypervigilance, which are known to affect executive, attentional and memory domains.

The study was conducted as a cross‐sectional analysis and included a carefully selected sample of 80 participants, comprising 41 patients diagnosed with chronic painful TMD and 39 healthy controls, matched by age, gender and sociodemographic characteristics (education, occupation and socioeconomic status), aged between 18 and 55 years, with pain‐related TMD for at least for 3 months. Patients were recruited from both the Temporomandibular Disorder and Orofacial Pain Service and the TMD at the School of Dentistry of Ribeirão Preto—University of São Paulo and University of Ribeirão Preto, over an 11‐month period, from March 2021 to February 2022. Control participants were recruited from the same academic and community environment as the TMD group, including students, staff and volunteers from the University of Ribeirão Preto and surrounding areas. Inclusion criteria for the control group included the absence of chronic orofacial pain, no clinical diagnosis of TMD according to the Diagnostic Criteria for Temporomandibular Disorders (DC/TMD), and age between 18 and 55 years. The same exclusion criteria used for the TMD group were applied to controls: history of neurological or psychiatric disorders, tumours, major surgeries involving the stomatognathic system within the past 6 months, or any condition that could compromise cognitive function or verbal communication. These criteria ensured the selection of healthy individuals for valid between‐group comparisons of cognitive performance. Each participant was invited by the same researcher, who personally approached them to explain the study objectives, procedures and expectations. This researcher was specifically trained and calibrated in the application of the DC/TMD and the MoCA, ensuring consistency and reliability in data collection and evaluation across all clinical assessments [15].

In addition to matching participants by age and gender, efforts were made to ensure similarity in educational level, occupation and socioeconomic status across groups. Educational attainment was recorded in total years of formal education and used to apply the standard correction for MoCA scores when participants had ≤ 12 years of schooling. Occupation was categorised based on self‐report into four groups: (1) university students, (2) employed professionals, (3) unemployed individuals and (4) retired. Socioeconomic status was assessed using self‐reported monthly household income and classified into three categories—low, middle and high—based on the criteria established by the Brazilian Economic Classification Criteria (Critério de Classificação Econômica Brasil, CCEB 2019, ABEP). These sociodemographic variables were collected using a structured questionnaire and were descriptively compared between the TMD and control groups to confirm their distributional similarity. Although formal statistical matching procedures were not employed, recruitment strategies were designed to approximate equivalent profiles between groups, minimising potential sociodemographic confounding.

The DC/TMD is a standardised and validated protocol designed for clinical and research purposes. The DC/TMD is structured in two axes: Axis I provides reliable diagnostic criteria for the most common TMD subtypes (such as myalgia, arthralgia and disc displacement), while Axis II assesses psychological distress and pain‐related disability. This instrument demonstrates strong psychometric properties, with inter‐examiner reliability ranging from *κ* = 0.63 to 0.85, and diagnostic sensitivity and specificity generally above 0.86 [15]. The Brazilian Portuguese version of the DC/TMD Axis I has also shown good validity and reliability, supporting its applicability in clinical research settings [16]. All evaluations were performed by a trained and calibrated examiner, following the standardised DC/TMD protocol. To minimise potential bias, the data analysis was conducted independently by two additional researchers who did not participate in the clinical evaluations. These analysts received anonymised data, allowing for objective interpretation and reducing the influence of subjective factors related to clinical interactions. The use of DC/TMD for diagnosing TMD followed established protocols, providing a standardised approach to accurately assess TMD symptoms.

The exclusion criteria were carefully applied to maintain sample consistency. Participants with a history of psychiatric or neurological disorders that could impair verbal communication or cognitive function, as well as those with tumours or major surgeries in the stomatognathic system within the past 6 months, were excluded from the study. These criteria helped ensure that the sample was homogenous in terms of cognitive and physical health, thereby strengthening the reliability of comparisons between the TMD and control groups.

### 2.4 Assessments

Cognitive performance was thoroughly assessed using the MoCA, a validated tool widely used to screen for mild cognitive impairments across multiple cognitive domains. The MoCA includes tasks that evaluate visuospatial/executive functions, attention, language, memory and orientation, providing a comprehensive overview of cognitive functioning in participants. Each participant completed the MoCA under the guidance of the trained researcher to ensure accurate scoring and consistency in administration.

Educational attainment was systematically recorded for all participants, given its well‐established influence on cognitive performance measures such as the MoCA. Although education level was not used as a formal matching criterion during group allocation, the distributions of years of education were similar between the TMD and control groups. To minimise potential bias and enhance comparability, we applied the standardised correction to the MoCA total score, adding one point for participants with 12 or fewer years of formal education, as recommended in the Brazilian Portuguese validated version of the instrument [18]. This correction was applied uniformly to all participants who met the criterion, and the adjusted scores were used in all statistical analyses. While educational level was not included as a covariate in the regression models, the correction procedure ensured that variations in education were accounted for in the primary outcome, thereby reducing the risk of confounding and strengthening the validity of between‐group comparisons.

Pain‐related factors were evaluated using three specific instruments. Pain intensity was measured through the Visual Analogue Scale (VAS), a reliable tool that allows participants to indicate their level of pain on a continuum, providing a quantifiable score for pain severity. Pain vigilance or the tendency to remain focused on pain signals, was assessed using the Pain Vigilance and Awareness Questionnaire (PVAQ). The PVAQ, validated for Brazilian Portuguese, consists of items that capture the participant's attention to and awareness of pain, offering insights into hypervigilant pain behaviours. Lastly, the Pain Catastrophizing Scale (PCS) was employed to measure catastrophising tendencies, including elements of helplessness, magnification and rumination, which reflect exaggerated negative thoughts and emotions about pain. The PCS is also validated for the Brazilian population and allows for a structured evaluation of participants' emotional responses to pain. Together, these instruments provided a multidimensional profile of each participant's cognitive function and pain‐related psychological responses, allowing for a detailed analysis of how cognitive impairments and pain perception interact in patients with chronic painful TMD.

### 2.5 Instruments

#### 2.5.1 Measurement of Cognitive Performance (MoCA Test)

The MoCA test (Montreal Cognitive Assessment) is a screening tool for mild cognitive impairments validated for Brazilian Portuguese and includes a one‐page set of tasks evaluating eight cognitive domains, which are measured through specific tasks, with scores assigned according to the respondent's performance, as described below (higher scores indicate better function): short‐term memory (delayed recall, 5 points); visuospatial skills (cube drawing, 1 point; clock drawing, 3 points); executive function (trail‐making test, 1 point; phonemic verbal fluency, 1 point; verbal abstraction, 2 points); attention, concentration and working memory (cancellation, 1 point; subtraction, 3 points; digit span, 2 points); language (naming, 3 points; sentence repetition, 2 points); and orientation to time (3 points) and space (3 points) [17, 18]. The sum of the scores obtained in the eight domains results in the total score, which can range from 0 to 30. Values below 26 indicate cognitive impairment. To correct for the educational effects noted in the original study, an additional point was given to individuals with 12 or fewer years of education [19]. The test was guided and administered by a previously trained researcher and the application time was approximately 13 min.

#### 2.5.2 Pain Vigilance and Awareness Questionnaire

To assess pain hypervigilance related to TMD, the PVAQ was self‐administered, which has been validated for Brazilian Portuguese [20]. It consists of 16 items, each containing statements about attention to and vigilance for pain. Respondents are required to select, according to a six‐point Likert scale (0 = ‘never’ to 5 = ‘always’), the option that best fits their perception, which will represent the degree of each pain‐related behaviour description. The higher the score, the greater the level of hypervigilance. As the purpose was to analyse the association of this independent variable with cognitive performance, only the total score was used.

#### 2.5.3 Pain Catastrophizing Scale (PCS‐BP)

The Brazilian Portuguese (BP)‐PCS protocol, the validated Brazilian version of the PCS, was used to measure pain catastrophising levels in the sample. This Scale has three dimensions (helplessness, magnification and rumination), each comprised of a set of items with statements that must be scored by the respondent according to their perception, following a Likert‐type scale that has a numerical range from zero (almost never) to four (all the time). The dimensions can be analysed separately or by adding the scores of all 13 statements, which results in a total level of pain catastrophising [20, 21].

### 2.6 Statistical Analyses

Before proceeding with group comparisons and examining relationships among variables, data distribution was evaluated using the Shapiro–Wilk normality test. This test was chosen for its sensitivity in detecting deviations from normality, particularly in smaller samples, providing a robust assessment of whether the data followed a normal distribution. With *p*‐values < 0.05, the test results indicated that the data met the assumptions of normality, allowing the use of parametric statistical tests. For the comparison of group means, independent *t*‐tests were conducted to determine significant differences between the TMD and control groups across various cognitive and pain‐related measures. This method enabled precise comparisons between the two independent groups, offering insights into whether patients with TMD exhibit distinct patterns in cognitive performance and pain responses compared to healthy controls.

Following these initial comparisons, multiple regression analysis was employed to explore the associations between specific cognitive domains and pain‐related factors, including pain intensity, hypervigilance and catastrophising. By including age and gender as covariates in the model, the regression analysis accounted for potential confounding effects, ensuring that observed relationships between cognitive function and pain‐related variables were not influenced by demographic differences. The inclusion of age and gender adjustments allowed for a more accurate interpretation of how pain‐related factors uniquely impact cognitive performance. No missing data were observed for the variables included in the analysis. To assess potential multicollinearity among the predictor variables—pain intensity, pain hypervigilance and pain catastrophising—we calculated the variance inflation factor (VIF) for each variable prior to conducting multiple regression analyses. All VIF values were below 2.0, indicating low collinearity and supporting the inclusion of these predictors within the same model. These diagnostics ensured the stability of the regression coefficients and the validity of the estimated associations. A significance level of 5% (*α* = 0.05) was applied to all analyses, balancing sensitivity to detect meaningful relationships with control over type I error rates. It was used the IBM SPSS Statistics 30.0.0.

## 3 Results

**FIGURE 1:**
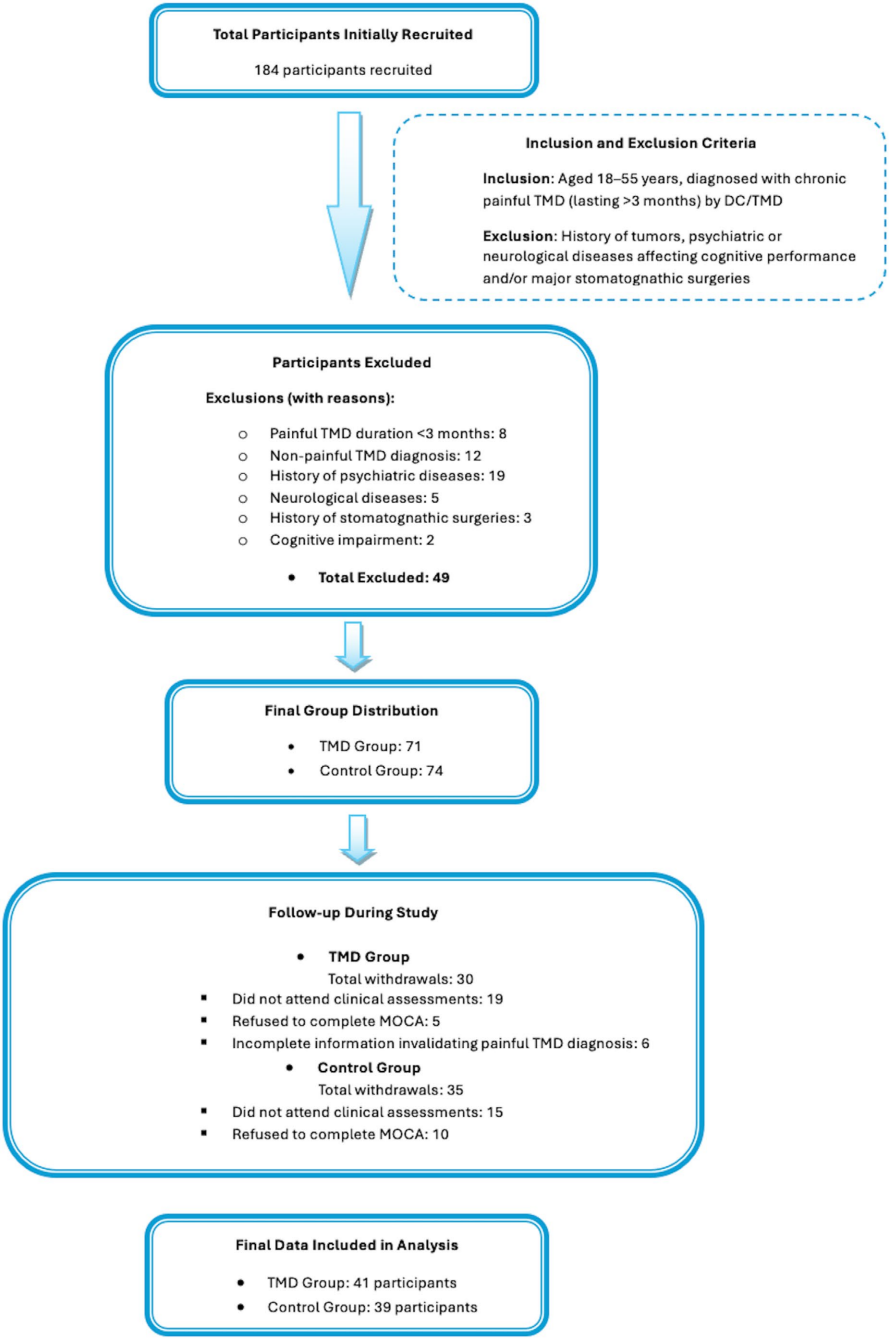
Flowchart of participant selection and retention in the study. This figure illustrates the inclusion and exclusion criteria applied to recruit participants for the study. The initial cohort consisted of 184 individuals, of whom 49 were excluded based on criteria such as a painful TMD duration of < 3 months, non‐painful TMD diagnosis or history of psychiatric, neurological or significant stomatognathic conditions. After exclusions, the TMD and control groups included 71 and 74 participants, respectively. A total of 30 participants from the TMD group and 35 from the control group withdrew during the study, resulting in final samples of 41 participants for the TMD group and 39 for the control group.

The flowchart in Figure 1 outlines the progression of participants through each phase of the study, starting from initial recruitment to final data analysis. A total of 184 participants were initially recruited, with 71 allocated to the TMD group and 74 to the control group after applying inclusion criteria (age 18–55 years and diagnosis of chronic painful TMD lasting more than 3 months) and exclusion criteria (history of tumours, psychiatric or neurological conditions affecting cognitive performance or major stomatognathic surgeries). Following this, 49 participants were excluded due to reasons such as non‐painful TMD diagnosis, duration of painful TMD under 3 months and significant medical histories. Additionally, participant follow‐up revealed 30 withdrawals from the TMD group and 35 from the control group, with reasons including non‐attendance at clinical assessments and refusal to complete the MOCA. Ultimately, 41 participants from the TMD group and 39 from the control group were included in the final analysis, ensuring a robust sample for assessing cognitive impacts associated with chronic painful TMD.

The descriptive analysis revealed notable differences between the TMD and control groups in some variables. Women comprised most participants in both groups, with 36 women in the TMD group and 22 in the control group. Age did not significantly differ between groups (*p* = 0.12), with women in the TMD group having a mean age of 36.9 years (±7.2) and men 34 years (±3), compared to 34.5 years (±6.7) for women and 37.2 years (±4) for men in the control group. However, significant differences were observed in cognitive performance, with the TMD group showing a lower mean MOCA score (25.1 ± 3.1) compared to the control group (28.4 ± 2.1, *p* = 0.03). The TMD group also reported a higher number of painful comorbidities (2.4 ± 1.1), while the control group reported none. Physical activity was significantly lower in the TMD group (2.1 ± 0.9) compared to controls (3.9 ± 1.2, *p* = 0.04). Although no significant difference was found in social life scores (*p* = 0.15), the TMD group demonstrated significantly higher scores on the PCS (34.9 ± 5.5) compared to the control group (19.7 ± 4, *p* = 0.001). These results suggest that patients with chronic painful TMD experience greater cognitive impairment and more catastrophic thoughts (Table 1).

**TABLE 1:** Descriptive statistics comparing cognitive and pain‐related variables between TMD and control groups. The table presents the mean (*M*) and standard deviation (SD) for age, total MOCA score, number of painful comorbidities, physical activity, social life activity, PVAQ and PCS scores for both TMD and control groups.

	TMD	Control	*p*
Gender	Women—36	Women—29	___
	Men—5	Men—10	
Age	Women—36.9 (±7.2)	Women—34.5 (±6.7)	
	Men—34 (±3)	Men—37.2 (±4)	0.12
MOCA—total score	25.1 (±3.1)	28.4 (±2.1)	0.03*
Visuospatial/Executive	3.6 (±0.6)	4.8 (±0.3)	0.03*
Attention	3.2 (±0.5)	5.9 (±0.5)	0.002*
Language	2.7 (±0.3)	2.9 (±0.1)	0.09
Abstraction	1.8 (±0.3)	2 (±0)	0.2
Memory	2.9 (±0.8)	4.7 (±0.4)	0.001*
Orientation	5.4 (±0.4)	5.8 (±0.2)	0.1
Number of painful comorbidities	2.4 (±1.1)	0	___
Physical activity	2.1 (±0.9)	3.9 (±1.2)	0.04*
Social Life	2.7 (±0.8)	3.1 (±0.6)	0.15
PVAQ	46.6 (±3)	___	___
PCS	34.9 (±5.5)	19.7 (±4)	0.001*

*Source:* own elaboration. Statistical comparisons between groups were performed using the independent *t*‐test. Significant differences are indicated by *p*‐values < 0.05 (indicated in bold text and with an asterisk).

Abbreviations: M, mean; MOCA, Montreal Cognitive Assessment; PCS, Pain Catastrophizing Scale; PVAQ, Pain Vigilance and Awareness Questionnaire; SD, standard deviation; TMD, temporomandibular disorder.

In the TMD group, participants were further classified according to their specific DC/TMD painful subdiagnoses. As shown in Table 2, the most frequent subtypes were myofascial pain with referral (*n* = 12) and mixed diagnoses involving both muscular and intra‐articular components (*n* = 12), followed by arthralgia (*n* = 9) and local myalgia (*n* = 8). Clinical profiles varied across subgroups. Participants with local myalgia exhibited the lowest scores in pain duration (10.6 ± 3.8 months), pain intensity (5.3 ± 1.0), hypervigilance (41.2 ± 3.0) and catastrophising (29.6 ± 4.5), suggesting a less severe pain‐related profile. In contrast, those in the mixed and myofascial pain with referral groups presented with higher pain‐related and psychosocial scores, reflecting a more complex clinical presentation.

**TABLE 2:** Clinical characteristics of painful TMD subdiagnoses according to DC/TMD.

Subdiagnosis (DC/TMD)	*N*	Pain duration (months)	Pain intensity (VAS 0–10)	Hypervigilance (PVAQ)	Catastrophising (PCS)
Local myalgia	8	10.6 ± 3.8	5.3 ± 1.0	41.2 ± 3.0	29.6 ± 4.5
Myofascial pain with referral	12	20.5 ± 5.9	7.1 ± 1.4	47.6 ± 2.8	36.1 ± 4.7
Arthralgia	9	14.8 ± 7.1	5.9 ± 1.6	44.3 ± 3.5	31.2 ± 6.0
Mixed (e.g., Myalgia + intrarticular TMD)	12	22.4 ± 5.2	7.3 ± 1.1	48.4 ± 2.6	37.5 ± 5.4

*Source:* Own elaboration. Values are expressed as Mean ± SD.

**FIGURE 2:**
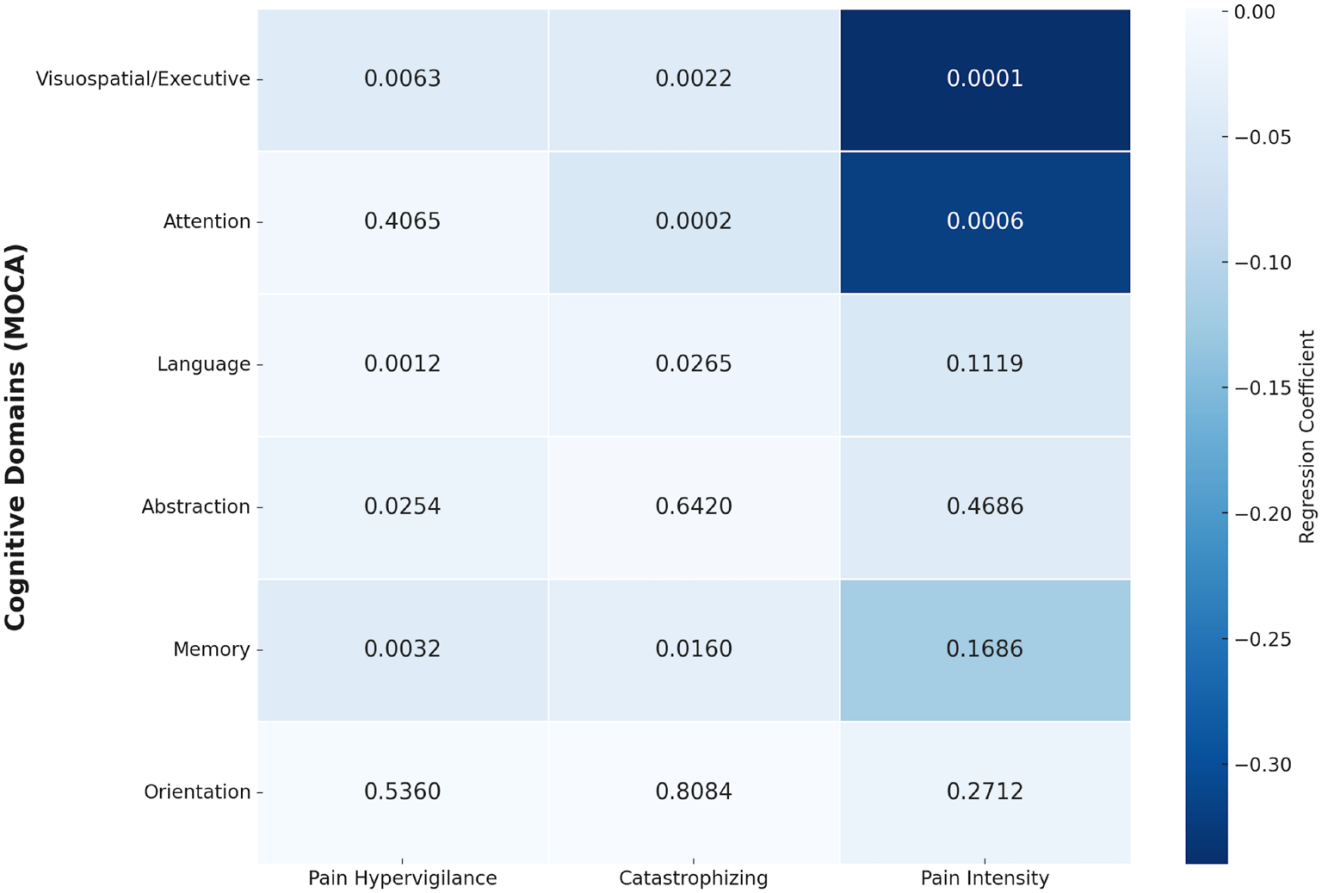
Heatmap of regression coefficients illustrating the associations between cognitive domains (assessed by MOCA) and pain‐related variables, including pain hypervigilance, pain catastrophising and pain intensity. The colour gradient represents the strength and direction of the relationship, with darker shades indicating stronger negative associations. The values within each cell represent the *p*‐values, indicating the statistical significance of each regression coefficient. The visuospatial/executive, attention and memory domains were notably impacted by pain‐related factors, suggesting specific cognitive impairments related to increased pain perception.

The regression analysis revealed significant associations between cognitive domains assessed by the MOCA and various pain‐related aspects, including pain hypervigilance, pain catastrophising and pain intensity. Overall, increased intensity of these pain‐related variables was associated with reduced performance in some cognitive domains (Figure 2). Notably, the visuospatial/executive domain demonstrated a particularly strong relationship with pain intensity, with a regression coefficient of −0.34 (95% CI: −0.50 to −0.18; *p* < 0.0001). This finding suggests that individuals experiencing higher levels of pain intensity tend to show significantly lower performance in visuospatial and executive functioning, emphasising the detrimental impact of pain on these cognitive abilities. Furthermore, significant negative associations were also observed between pain hypervigilance and the visuospatial/executive domain, with a regression coefficient of −0.04 (95% CI: −0.06 to −0.01; *p* = 0.0063), indicating that increased hypervigilance to pain is also linked to reduced visuospatial and executive skills.

Additionally, pain hypervigilance and catastrophising were negatively correlated with other cognitive domains, such as attention and language. The regression coefficient between pain catastrophising and attention was −0.05 (95% CI: −0.08 to −0.02; *p* = 0.0002), suggesting a substantial relationship between higher levels of catastrophising and reduced attentional capacity. For the language domain, both pain hypervigilance and catastrophising showed significant negative correlations, with coefficients of −0.01 (95% CI: −0.02 to −0.01; *p* = 0.0012) and −0.015 (95% CI: −0.02 to −0.001; *p* = 0.0265), respectively. These results imply that heightened awareness and exaggerated negative thoughts about pain are linked to poorer language performance. Moreover, for the memory domain, pain hypervigilance was found to have a significant negative relationship (coefficient = −0.04; 95% CI: −0.06 to −0.01; *p* = 0.0032) (Figure 2).

**FIGURE 3:**
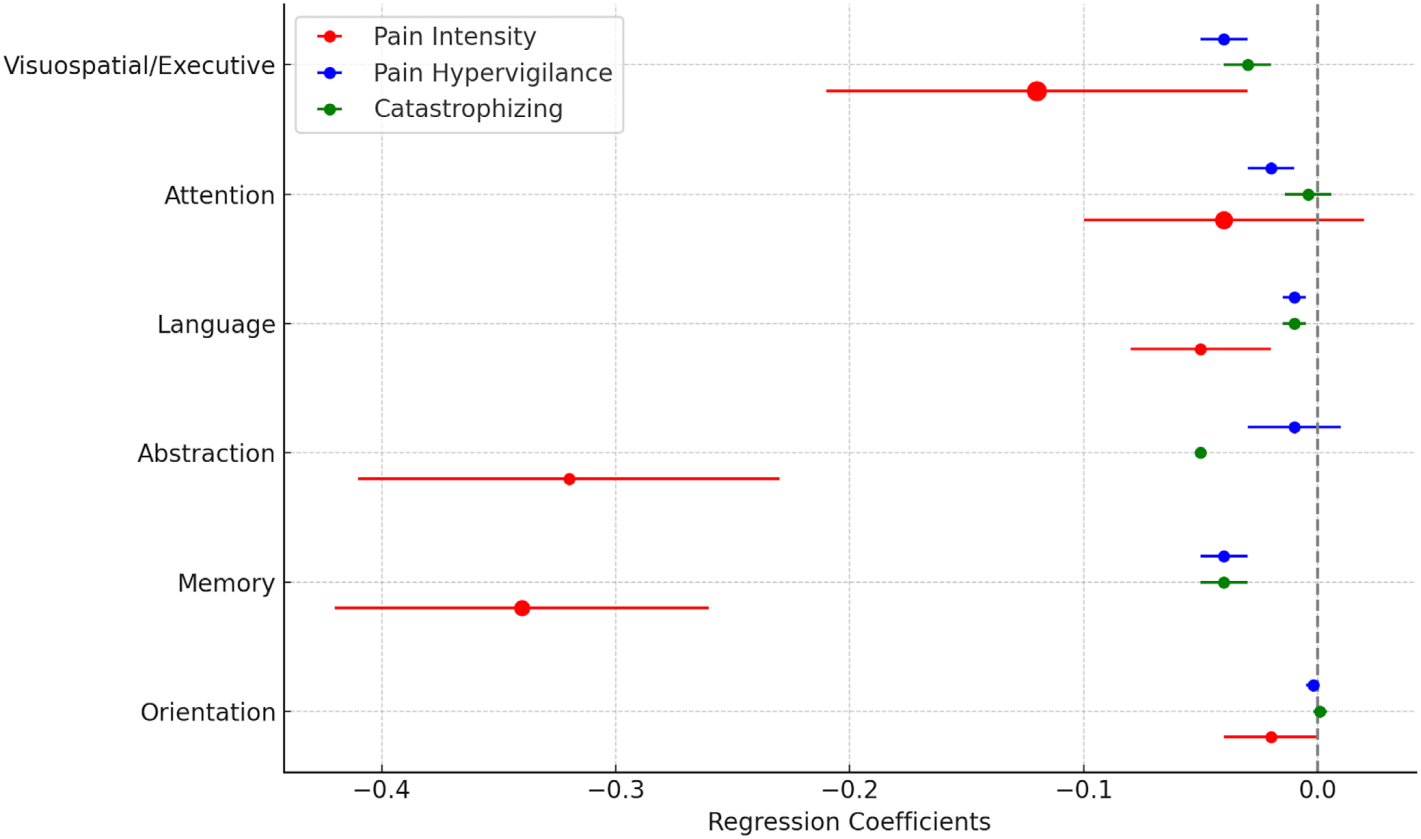
Dot plot illustrating the regression coefficients and standard errors for pain hypervigilance, catastrophising and pain intensity across six cognitive domains: Orientation, memory, abstraction, language, attention and visuospatial/executive. Larger markers indicate domains more strongly influenced by pain intensity, with significant associations (*p* < 0.05) observed particularly in the visuospatial/executive and attention domains. Smaller markers represent weaker associations, such as in the abstraction domain. The vertical dashed line represents the null value (coefficient = 0), indicating no effect.

Figure 3 presents a dot plot illustrating the regression coefficients for pain hypervigilance, catastrophising and pain intensity across MOCA domains. The coefficients reflect the strength and direction of the association between each pain variable and the cognitive domains. Larger markers are used to highlight domains more strongly influenced by pain intensity, such as visuospatial/executive and attention, where significant associations were observed (*p* < 0.05). Notably, domains like abstraction showed weaker associations, as indicated by smaller marker sizes. These findings suggest that cognitive functioning, particularly in the visuospatial and attentional domains, may be more susceptible to pain‐related factors.

**FIGURE 4:**
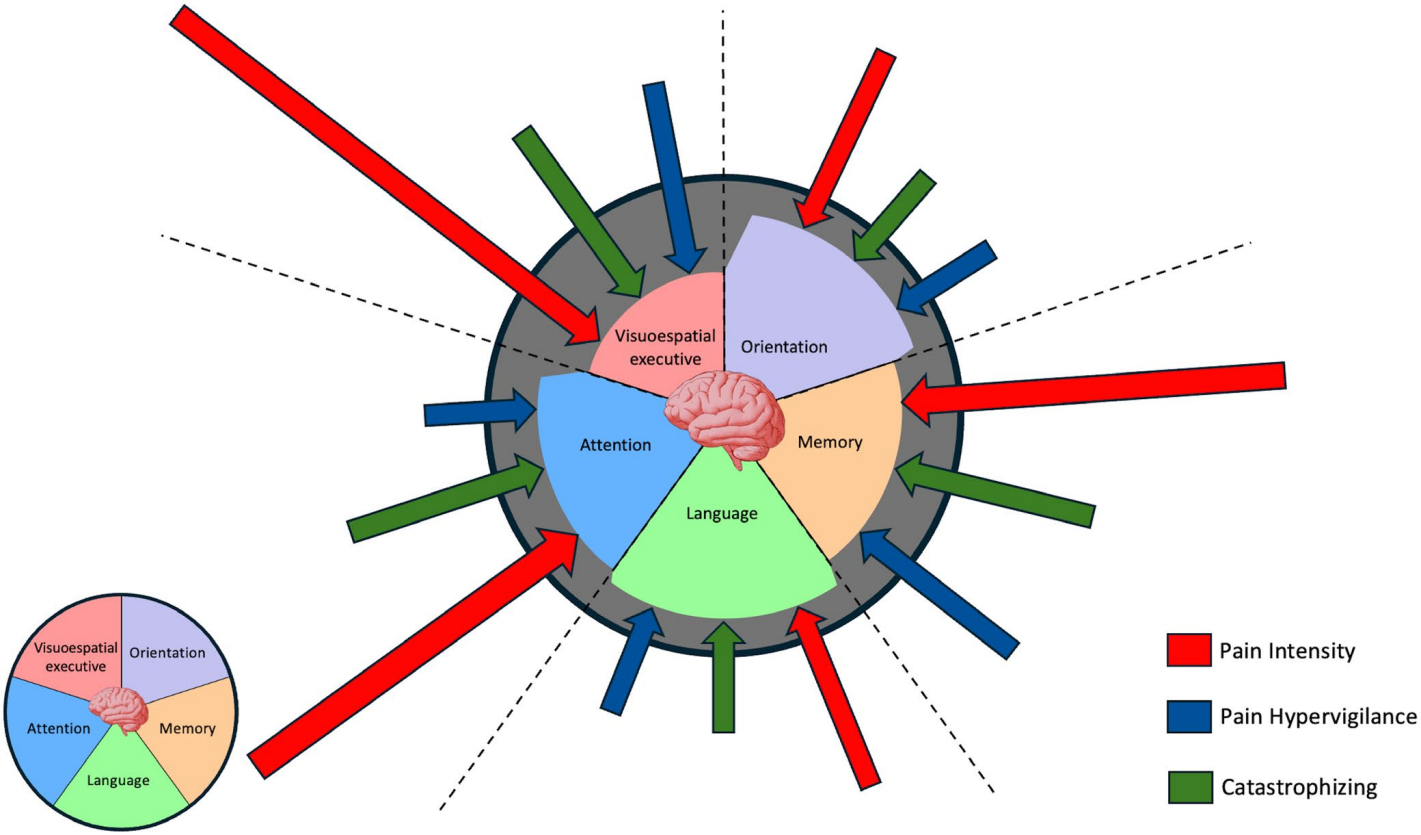
Associations between cognitive domains and pain‐related factors in chronic painful TMD patients. The diagram illustrates the impact of pain intensity (blue), catastrophising (orange) and pain hypervigilance (green) on various cognitive domains, including visuospatial/executive functioning, orientation, memory, language and attention. The thickness of the arrows represents the strength of each association, with thicker arrows indicating stronger relationships. Pain intensity shows a prominent association with visuospatial/executive and attention domains, while catastrophising is primarily linked to attention and language impairments, and hypervigilance is associated with memory and orientation deficits. These findings emphasise the widespread effect of pain‐related factors on cognitive performance in chronic TMD.

Figure 4 illustrates the associations between cognitive domains and pain‐related factors—pain intensity, catastrophising and hypervigilance—as identified in our study. Each cognitive domain (visuospatial/executive, orientation, memory, language and attention) is impacted by varying levels of these pain‐related variables. Arrows of different colours and sizes represent the strength and direction of these associations, with thicker arrows indicating stronger correlations. Pain intensity, shown in blue, demonstrated the most substantial impact, particularly on the visuospatial/executive and attention domains, suggesting that heightened pain perception impairs cognitive performance in these areas. Catastrophising, represented in orange, was significantly linked to reduced attentional capacity and language performance, while hypervigilance, in green, was notably associated with impairments in memory and orientation. This visual model underscores the pervasive influence of pain on multiple cognitive functions, highlighting the need for comprehensive treatment approaches that address both cognitive and pain‐related factors in chronic TMD patients. No subgroup or interaction analyses were conducted as they were not within the scope of this study.

## 4 Discussion

This study demonstrated that individuals with chronic painful TMD presented significant impairments in specific cognitive domains, most notably in visuospatial/executive functioning, attention and memory, when compared to healthy controls. These deficits were not uniform but rather domain‐specific, suggesting that chronic pain does not cause a global cognitive decline but affects particular cognitive processes. Among the psychological factors related to pain, elevated levels of pain intensity and pain catastrophising emerged as the most relevant predictors of reduced cognitive performance, especially in domains requiring executive control and sustained attention. Hypervigilance was also significantly associated with poorer performance in memory and language tasks, reinforcing the hypothesis that excessive attentional focus on pain‐related stimuli may interfere with cognitive resource allocation. These findings support the view that psychological dimensions of pain play a central role in modulating cognitive function in TMD and may contribute to functional limitations in daily life. Such effects may be partially explained by the overlap between the neural circuits involved in pain processing—particularly those in the prefrontal cortex and limbic system—and those responsible for cognitive functions such as attention, memory and executive control [5, 6, 12, 14, 22]. Our findings align with previous studies that have used the MoCA to assess cognitive dysfunction in other chronic pain populations, such as fibromyalgia, chronic low back pain and migraine. These studies consistently report impairments in attention, executive functioning and memory domains—patterns similar to those observed in our TMD sample [4, 10, 12]. While the underlying mechanisms may differ across conditions, the convergence of cognitive deficits reinforces the relevance of using domain‐specific hypotheses when evaluating chronic pain populations.

The findings revealed that higher levels of pain catastrophising were significantly associated with reduced attention and language performance, suggesting that maladaptive pain‐related cognitions can exert a substantial negative impact on cognitive functioning. These results are consistent with recent evidence indicating that chronic pain conditions—especially those marked by emotional dysregulation, negative affectivity and cognitive overload—can compromise attentional efficiency and verbal fluency [10, 13]. Pain catastrophising is characterised by persistent negative thoughts about pain, feelings of helplessness and magnification of perceived threat, which are known to intensify emotional arousal and stress reactivity. These mechanisms may lead to the hyperactivation of limbic structures and prefrontal cortical regions involved in attentional control and language processing [13]. Additionally, prolonged exposure to maladaptive cognitive‐emotional states, such as catastrophising, has been linked to neuroinflammatory activity and suppressed neurogenesis, particularly in the hippocampus and prefrontal cortex, contributing to impairments in working memory and cognitive flexibility [4, 10, 13]. In light of these findings, our results reinforce the hypothesis that pain catastrophising does not merely reflect an emotional response to pain, but actively contributes to domain‐specific cognitive impairments. It increases the cognitive load and disrupts neural circuits fundamental to executive and attentional regulation, making it a key target for cognitive and behavioural interventions in chronic pain management.

The most notable finding of this study was the robust association between pain intensity and deficits in visuospatial/executive functioning and attention. Patients reporting higher levels of pain intensity exhibited significant impairments in these cognitive domains, suggesting that the perceptual and affective dimensions of pain may directly interfere with cognitive efficiency. This relationship may be explained by the shared neurocognitive pathways involved in pain modulation and executive processing, particularly within the prefrontal cortex. Furthermore, hypervigilance to pain—defined as an increased and persistent attentional focus on pain‐related stimuli—was found to significantly affect memory and language abilities, likely due to the continuous allocation of cognitive resources to pain monitoring at the expense of other mental operations. Catastrophising also contributed to reductions in attention and memory, underscoring its central role in the cognitive burden experienced by individuals with chronic pain. Together, these findings suggest that cognitive deficits in patients with chronic painful TMD are not uniform but rather linked to specific psychological and perceptual pain dimensions, especially in domains requiring executive control, sustained attention and working memory. The tendency to ruminate on current pain or to anticipate future pain states may reinforce maladaptive neural plasticity while weakening circuits responsible for adaptive cognitive responses [5, 13, 14, 22]. These results further validate the use of domain‐specific cognitive hypotheses in studies of chronic pain and highlight the need for integrative assessment models that include cognitive screening alongside traditional pain measures.

Our findings contribute to a growing body of literature that underscores the cognitive repercussions of chronic pain conditions [5, 11, 12, 13, 22]. In line with previous studies, our results suggest that pain‐related factors extend beyond physical discomfort and are intricately linked to various aspects of cognitive functioning [6]. This study enriches our understanding of these associations by providing insights into how specific pain‐related perceptions impact distinct cognitive domains, underscoring the complexity of chronic pain's effect on cognitive performance.

The most notable finding in our analysis was the robust association between pain intensity and deficits in visuospatial/executive functioning and attention. Patients with heightened pain intensity displayed particularly pronounced impairments in these areas, reinforcing previous findings that chronic pain interferes with higher order cognitive processes [5, 23, 24]. Executive functioning and visuospatial abilities are critical for goal‐directed behaviour, cognitive flexibility and information integration. When these domains are compromised, individuals may face increased difficulty in performing everyday tasks, managing social roles and engaging in adaptive coping strategies. These impairments may be the result of shared neural circuits between pain modulation and cognitive control, particularly within the prefrontal cortex, which is heavily involved in both processes [5]. Chronic pain may overload these circuits, reducing the availability of cognitive resources necessary for planning, decision‐making and complex problem‐solving. This highlights a potential neurofunctional mechanism through which persistent nociceptive input contributes to executive dysfunction, reinforcing the importance of addressing pain intensity not only for symptom relief but also for preserving cognitive integrity.

Additionally, our findings reveal that patients experiencing higher levels of pain intensity showed significant reductions in cognitive domains, especially visuospatial/executive functions and attention. This observation suggests a direct impact of heightened pain perception on cognitive performance, supporting the hypothesis that chronic pain alters neural architecture and processing efficiency via mechanisms such as maladaptive neural plasticity, central sensitisation and dysregulation of pain modulatory systems [5, 25, 26, 27]. These changes may impair synaptic connectivity and functional networks responsible for attentional control and executive regulation. The brain's increased demand to process and regulate chronic pain may consume substantial cognitive resources, leaving fewer resources available for other tasks—a concept known as resource depletion [27]. For individuals with TMD, these deficits may further impair occupational, academic and interpersonal functioning, ultimately affecting overall quality of life. Thus, the cognitive impact of chronic pain should be considered a central feature of its burden, rather than a secondary consequence.

Furthermore, our analysis demonstrated that hypervigilance to pain significantly affected memory and language abilities, while catastrophising was associated with diminished attentional performance. Hypervigilance likely amplifies cognitive deficits by directing sustained and selective attention toward pain‐related cues, disrupting normal cognitive processing and reducing the availability of working memory resources. This persistent monitoring of somatic signals may impair memory encoding and retrieval processes, particularly in emotionally salient contexts. Likewise, catastrophising—which involves rumination, magnification and feelings of helplessness—can exacerbate attentional impairments through increased emotional arousal, stress reactivity and anxiety, all of which are known to interfere with executive attentional networks [11, 12, 28]. These findings align with growing evidence that psychological components of pain have measurable effects on cognitive function. The observed associations support the view that pain is not only a sensory experience but also a complex cognitive‐affective phenomenon, wherein maladaptive psychological responses—such as excessive worry or negative interpretation of symptoms—can disrupt cognitive domains selectively [29, 30]. As such, integrating cognitive screening and psychological assessment into chronic TMD management could improve both pain outcomes and cognitive well‐being.

Given the psychological profile of patients with chronic TMD—particularly their tendency toward catastrophising pain‐related thoughts and exhibiting hypervigilance regarding pain—it is common for these individuals to engage in the unsupervised use and misuse of medications to alleviate their pain. The frequent presence of comorbidities in this population often necessitates the use of additional medications. Although this study did not control for the confounding variable of ‘medications’, it is important to consider it as a potential factor that may interfere with cognitive aspects.

Taken together, these findings highlight that cognitive deficits in chronic TMD patients are closely related to the psychological and perceptual aspects of pain, especially within domains crucial for executive control, attention and memory. The interplay between pain perception and cognitive function observed in this study suggests that chronic TMD patients may benefit from multidisciplinary approaches that address not only the physical but also the cognitive and psychological dimensions of pain. Interventions such as cognitive‐behavioural therapy and mindfulness‐based therapies, which have been shown to reduce pain catastrophising and hypervigilance, may hold promise in alleviating these cognitive impairments and improving overall patient outcomes [6, 31, 32].

The findings associated with this study demonstrated that pain catastrophising was strongly associated with reduced attention and language performance, while hypervigilance correlated with memory deficits—highlighting the cognitive impact of maladaptive pain‐related processes. Cognitive‐behavioural therapy is a structured psychological intervention aimed at identifying and modifying dysfunctional beliefs and behaviours related to pain, which has been shown to reduce pain catastrophising and enhance attentional control [33]. Similarly, mindfulness‐based approaches, such as Mindfulness‐Based Stress Reduction (MBSR), emphasise focused attention and nonjudgmental awareness of present experiences, which can attenuate hypervigilance, reduce cognitive load and restore attentional and emotional balance [34, 35]. These interventions directly target the psychological constructs most strongly associated with cognitive dysfunction in our study, supporting their relevance as adjunctive strategies in the management of chronic TMD.

A strength of the present study is its potential contribution to advancing the clinical understanding of chronic painful TMD through the identification of specific cognitive domains impacted by pain‐related psychological factors. By demonstrating associations between pain catastrophising and attentional deficits, as well as between hypervigilance and memory impairment, the findings offer novel evidence that cognitive dysfunction in TMD is not diffuse, but domain‐specific. This differentiation enhances the clinical relevance of our results, as it underscores the importance of integrating cognitive screening into the multidisciplinary assessment of TMD patients. Moreover, these findings support the application of targeted therapeutic strategies—such as cognitive‐behavioural or mindfulness‐based interventions—that address not only pain‐related distress but also cognitive impairments that may exacerbate functional limitations. Such an approach aligns with contemporary biopsychosocial models of chronic pain management and may contribute to more individualised and effective care pathways.

Limitations include the cross‐sectional design, which precludes causal inferences, and the predominance of women in the sample, limiting generalisability. Future longitudinal studies with diverse populations should explore the temporal relationships between pain‐related factors and cognitive impairments. The predominance of women in our sample, while reflective of TMD prevalence, may overestimate or underestimate gender‐specific cognitive effects. Additionally, the reliance on self‐reported measures may amplify emotional biases linked to catastrophising or hypervigilance, potentially magnifying associations with cognitive domains. Finally, while the MOCA provides a broad assessment of cognitive domains, more specific neuropsychological tests could offer a more nuanced understanding of cognitive impairments in this population. Future research addressing these limitations could further elucidate the complex relationship between chronic pain and cognitive function in TMD. While our findings align with existing literature, the multiplicity of analyses conducted in this study necessitates cautious interpretation to avoid overestimating the strength of the associations observed.

Another important limitation to consider is the potential for residual confounding due to qualitative differences in educational background between the groups. Although a standard correction was applied to MoCA scores for participants with 12 or fewer years of formal education, this adjustment may not fully account for disparities in the quality and context of educational experiences. Notably, the control group consisted largely of university students and staff, who may be exposed to more cognitively enriching environments compared to the clinical TMD group. These contextual differences could systematically favour higher cognitive performance in the control group, independent of pain‐related factors. Therefore, caution is warranted when interpreting between‐group differences, as subtle educational and environmental influences may persist beyond the standardised correction.

In conclusion, our study reveals that chronic painful TMD is associated with significant cognitive impairments, particularly in visuospatial/executive functioning, attention and memory. Elevated pain intensity and catastrophising emerged as the most impactful factors, closely linked to deficits in these cognitive domains. Additionally, pain hypervigilance contributes to impairments in memory and language, suggesting that various dimensions of pain perception exert distinct effects on cognitive processes. While our findings align with existing literature, the multiplicity of analyses conducted in this study necessitates cautious interpretation to avoid overestimating the strength of the associations observed.

## Author Contributions

This study was conceptualised and designed by J.R.‐M., M.H.F. and L.V.M., who also provided guidance throughout the research process. The experiments and data collection were carried out by J.R.‐M., L.G.A., C.B. and M.O.M., with significant contributions to the organisation and execution of the study protocols. The data analysis and interpretation of results were performed collaboratively by J.R.‐M. and L.G.A., ensuring a rigorous and thorough examination. The manuscript was primarily drafted by J.R.‐M., with substantial input and critical revisions provided by M.H.F. and L.V.M. to ensure clarity, coherence and alignment with the study's objectives. All authors actively contributed to discussions on the results and implications of the study, reviewed and approved the final version of the manuscript and agreed to be accountable for all aspects of the work, ensuring its accuracy and integrity.

## Conflicts of Interest

The authors declare no conflicts of interest.

## Data Availability

The datasets generated and/or analysed during the current study are available from the corresponding author on reasonable request.
